# Supplementary material for: Overexpression of the Bacteriophage T4 motB Gene Alters H-NS Dependent Repression of Specific Host DNA
Source: Viruses. 2021 Jan 9;13(1):84. doi: 10.3390/v13010084 (PMC7827196; doi:10.3390/v13010084)
Supplement: Supplementary file 1 [file viruses-13-00084-s001.zip › supplemental/Viruses Supplemental Material.pdf]

Table S1. Sequences of primers used to generate DNA PCR products. (See accompanying Table S1-S6.xlsx file, Table S1.) Product size indicates the length of PCR product produced using either the P_{I8} or P_{proV} primer sets.

Table S2. List of *E. coli* BL21(DE3) genes significantly up-regulated by MotB. (See accompanying Table S1-S6.xlsx file, Table S2.) RNA-seq analyses were performed as described in Materials and Methods and the RNA-seq data was processed using *E. coli* BL21(DE3) (NC_012971.2) as the reference genome. Column A indicates the locus tag for the gene. Differential expression between conditions (cells expressing *motB* versus cells containing the vector) was represented as the fold change, and genes with a fold change ≥ 2 (column B), mean reads (column C for cells expressing motB, column D for cells with the vector) ≥ 5, and an adjusted p-value (column E) ≤ 0.05 were considered significant. Genes (column F for name, column G for function) that stem from the λ DE3 lysogen or prophages are indicated in column H in red, while genes that stem from the host are in black. References in column I refer to previous reports of H-NS regulation of the gene. RNA-seq data is available in the National Center for Biotechnology Information (NCBI) database (GEO # GSE152170).

Table S3. Top 10 HHpred hits for MotB. (See accompanying Table S1-S6.xlsx file, Table S3.) Columns indicate: A) Hit rank; B) Protein PDB designation and chain name of the hit; C) Name of the protein; D) Function annotation according to HHpred; E) SCOP classification of the protein domains; F) Pfam family name of hit and the corresponding region within the protein; G) Species; H – M) values from HHpred analysis [1] indicating probability, E-value, P-value, Score, Identity, and Similarity, respectively; N) Region in MotB that aligned to the hit; O) Region in hit that aligned to MotB; P) HMM value of the pairwise comparison.

Table S4. MotB homologs found by PSI-BLAST. (See accompanying Table S1-S6.xlsx file, Table S4.) Homologs were identified as described in Materials and Methods. Columns indicate: A) NCBI Accession number of the protein; B) Protein name as indicated in RefSeq; the list order is the same as in the MotB phylogenetic tree shown in Figure 2, and the colors denote the branches shown in that tree (Category 1, magenta; Category 2, blue; Category 3, grey; Category 4, green); C- E) Taxonomy of the phage: Species, Genera, and Subfamily, respectively, with the Species and Genera columns colored as follows: *Tequatrovirus* in light orange, *Gaprivervirus* in light blue, *Dhakavirus* in beige, *Mosigvirus* in light green, unclassified *Tevenvirinae* in gray, and bacteria in no color. F - K). Statistics of PSI-BLAST results: Maximum Score [the highest alignment score of a set of aligned segments from the same subject (database) sequence], Total Score [sum of alignment scores of all segments from the same database sequence that match the query sequence (calculated over all segments)], Query Coverage (the percentage of query sequence that is covered by the target sequence), E value (the number of hits one can expect to see by chance when searching a database of a particular size), and Percentage of Identity (the percentage to which two sequences have the same residues at the same positions in an alignment), respectively.

Table S5. Description of MotB homologs annotated as bacterial genes. (See accompanying Table S1-S6.xlsx file, Table S5.) Columns indicate: A) Accession number of protein in NCBI Genbank; B) Protein name as indicated in RefSeq; C) Bacterial species; D – H) Statistics of PSI-BLAST results (see legend to Table S4 for more details): Maximum Score sequence, Total Score, Query Coverage, E value and Percentage of Identity, respectively; I) Strain; K) Refseq assembly identification number; L) Indicated geographical location of the sequenced biosample; M) Indicated source of the biosample; N) Further information about biosample.

Table S6. Number of MotB homologs found within phage. (See accompanying Table S1-S6.xlsx file, Table S6.) Columns indicate: A) Phage name; B) Total number of MotB homologs identified within that genome; C) Number of homologs within Category 1 (MotB branch of tree in Figure 2); D) Number of homologs within Categories 2-4 (MotB.1 branches of tree in Figure 2); E) Subfamily; F) Genera. The colors of the Genera are the same as in Table S4.

-

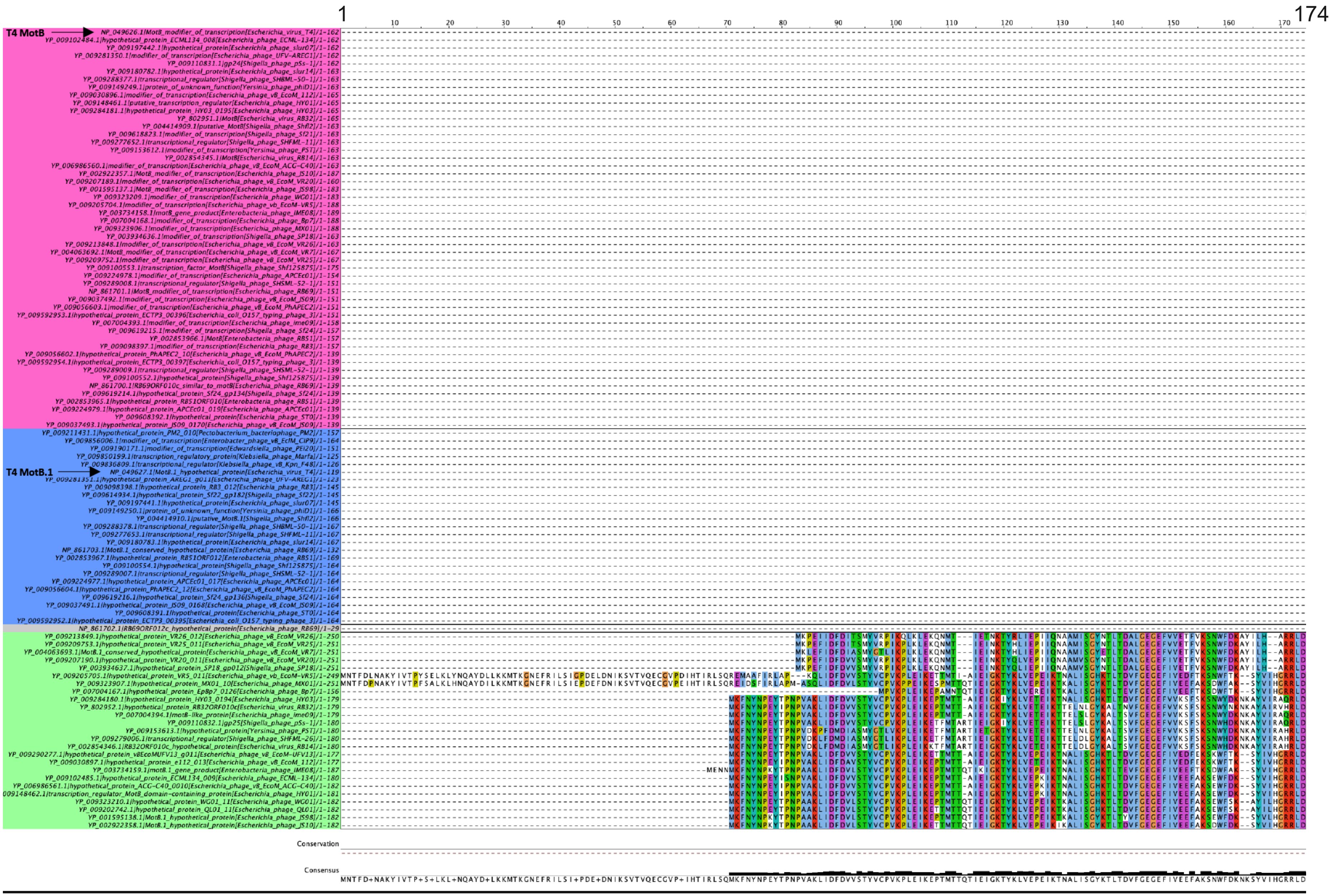
1
174
T4 MotB
T4 MotB.1
Conservation
Consensus

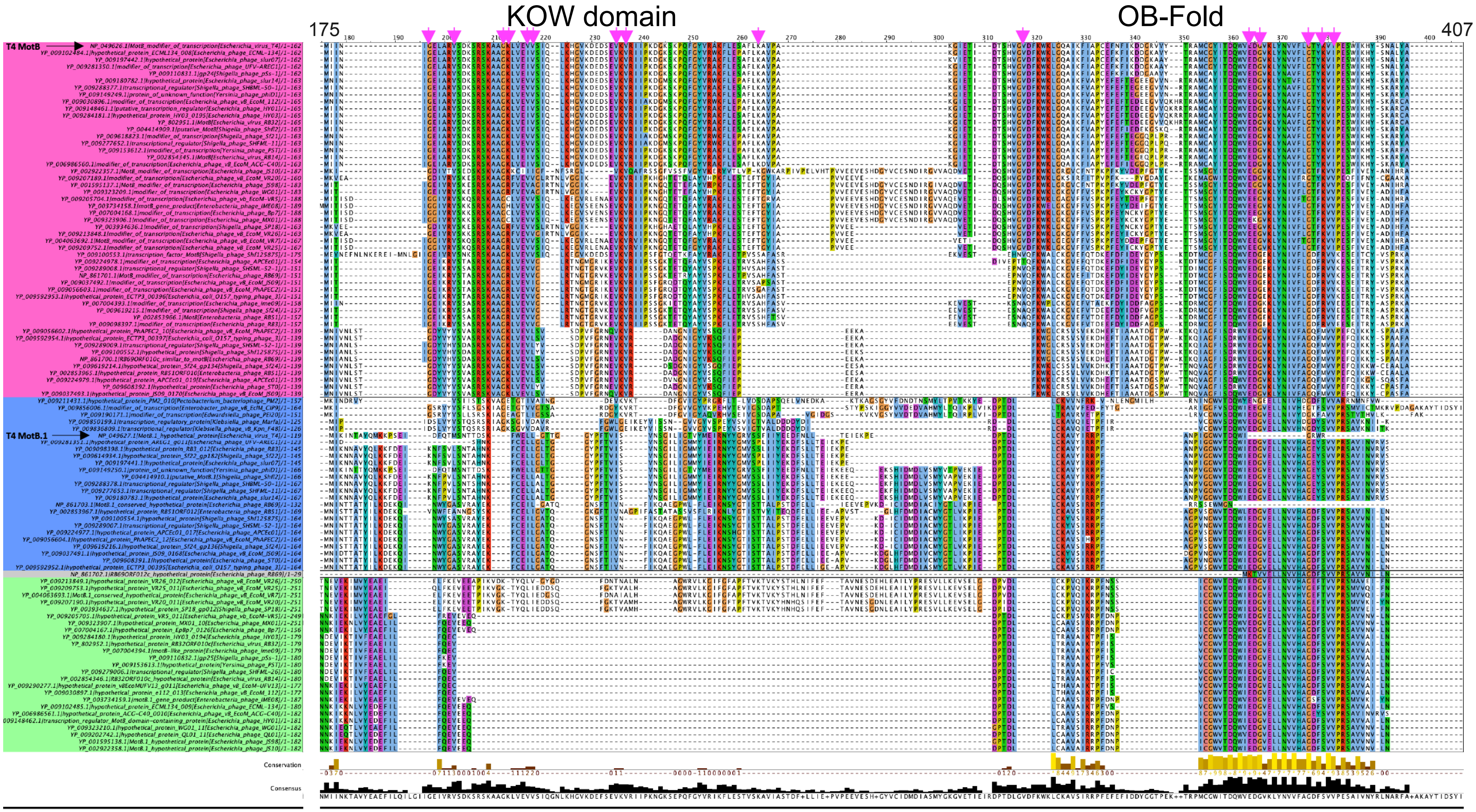

Figure S1. Multiple sequence alignment of MotB and MotB.1. The 102 phage PSIBLAST hits for T4 MotB were aligned using COBALT with manual gap adjustment. The sequences were reordered according to the UPGMA tree, and their names were colored according to the category branches in Figure 2 (Category 1, magenta; Category 2, blue; Category 3, grey; Category 4, green). Residues are colored using the CLUSTAL X scheme (blue, hydrophobic; red, positive charge; magenta, negative charge; green, polar; pink, cysteine; orange, glycine; yellow, proline; cyan, aromatic). Top panel shows residues 1-174; bottom panel shows residues 175-407. The large magenta arrows on top of the sequences in the bottom panel indicate the conserved residues in the KOW domain and the OB-fold

motifs as shown in Figure 1. The Conservation score display (below the alignment as a histogram) is the Alignment Conservation Annotation, a numerical index reflecting the conservation of physico-chemical properties of each column. Consensus at the bottom indicates the percentage of the particular residue per column. A"+" symbol means it is shared by more than one residue at that position. This figure was generated by JalView [2]

A.

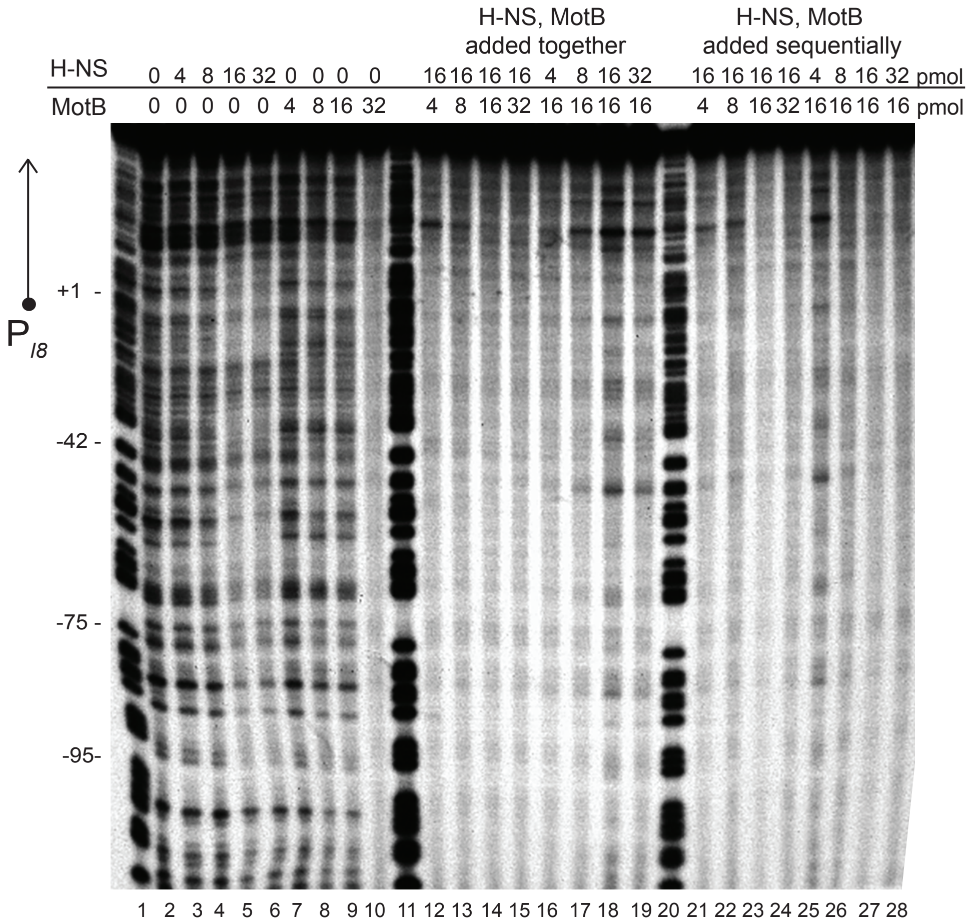

B.

```
    −140       −130       −120       −110
CTT CTTCCCGCTG GAAATCTGAC GTTATTCGTT TTGTTCATCC

−100       −90        −80        −70        −60
AGTAGGATTT GGATTTATAG CAATTACCCT TTTAACAATG TTTATTAATG

−50        −40        −30        −20        −10
TTGGTTTAAC TCTTAAACAT ACAGAGACTA TAATTAATAA ATACAAAAAC

→              +10        +20        +30        +40        +50
TATAAATGGG ATTCTGGATT GCCTACTGAA TATGCCGACA GAATAGCTAA

           +60        +70
ATTAACTCCA ACCGGTGAAA TTGAG
```

Figure S2. Representative DNase I footprints of MotB and/or H-NS at T4 P_{l8}. (A) DNA surrounding the P_{l8} promoter (positions -143 to +75; 0.05 pmol DNA; 10.9 pmol total bp; 5′-^{32}P labeled on nontemplate strand) was incubated with the indicated amount of MotB-His and/or H-NS and treated with DNase I before electrophoresis on a 5% (*w*/*v*) polyacrylamide, 7 M urea denaturing gel. A schematic of the P_{l8} promoter region is shown to the left of the gel and positions corresponding to the DNA sequence determined from the G + A ladder are indicated. (A) Lanes 1, 11, 20: G + A ladder; lane 2: no protein control; lanes 3-6: 4 pmol, 8 pmol, 16 pmol, and 32 pmol H-NS, respectively; lanes 7-10: 4 pmol, 8 pmol, 16 pmol, and 32 pmol MotB, respectively; lanes 12-15 and 21-24: 16 pmol H-NS with 4 pmol, 8 pmol, 16 pmol, and 32 pmol MotB, respectively; lanes 16-19 and 25-28: 16 pmol of MotB with 4 pmol, 8 pmol, 16 pmol, and 32 pmol H-NS, respectively. For lanes 12-19, H-NS and MotB were added together; for lanes 21-28, the protein whose concentration did not change was added first and incubated with the DNA for 10 min at 37° C before the addition of the second protein. (B) Nontemplate sequence surrounding P_{l8} (-143 to +75). The T4 late promoter TATA box is boxed, and the +1 TSS is indicated by the black arrow and larger font.

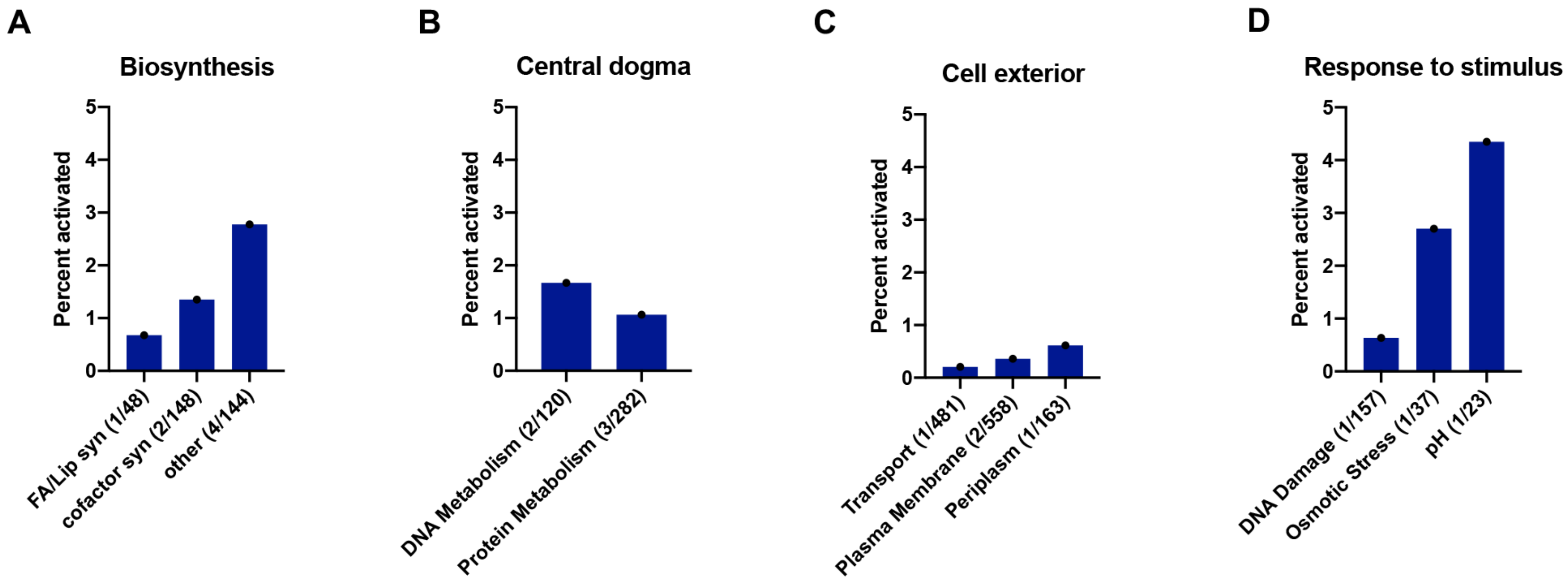

Figure S3. Visualization of transcriptomics data in representative categories. Bars represent percentages of genes within the corresponding host pathway increased by MotB production. The number of genes affected by MotB per total genes in the category is indicated with its name on the x-axis. Panels show the EcoCyc dashboard categories: (A) “Biosynthesis”, (B) “Central dogma”, (C) “Cell exterior”, and (D) “Response to stimulus”.

1. Soding, J.; Biegert, A.; Lupas, A. N., The HHpred interactive server for protein homology detection and structure prediction. *Nucleic Acids Res* **2005,** 33, (Web Server issue), W244-8.
2. Waterhouse, A. M.; Procter, J. B.; Martin, D. M.; Clamp, M.; Barton, G. J., Jalview Version 2--a multiple sequence alignment editor and analysis workbench. *Bioinformatics* **2009,** 25, (9), 1189-91.
